# Bridging continents: postgraduate infectious diseases training programs from central Europe to Southeast Asia

**DOI:** 10.1007/s15010-025-02597-7

**Published:** 2025-07-08

**Authors:** Selcen Öncü, Hakan Erdem, Zeliha Kocak Tufan, Seif Salim Al-Abri, Muna Al Maslamani, Jamal Wadi Alramahi, Sinan Alrifai, Ahmed Alsuwaidi, Altaf Ahmed, Rusmir Baljic, Bojana Beović, Rok Civljak, Amangul Duisenova, Dilruba Garashova, Krsto Grozdanovski, Arjan Harxhi, Tiberiu Holban, Souha Kanj, Suresh Kumar, Ainura Kutmanova, Masoud Mardani, Ziad Ahmed Memish, Egídia Gabriela Miftode, Sadie Namani, Serkan Öncü, Michael M. Petrov, Tomislav Preveden, Natalia Pshenichnaya, Bilal Ahmad Rahimi, Abdurashid Oblokulov, Yesim Taşova, Sotirios Tsiodras, George M. Varghese

**Affiliations:** 1 Department of Medical Education, Faculty of Medicine, Aydın Adnan Menderes University, Aydın, Turkey; 2 Department of Infectious Diseases and Clinical Microbiology, Turkish Health Sciences University, Gülhane School of Medicine, Ankara, Turkey; 3 Department of Infectious Diseases and Clinical Microbiology, Faculty of Medicine, Ankara Yıldırım Beyazıt University, Ankara, Turkey; 4 The Royal Hospital, Muscat, Oman; 5 Division of Infectious Diseases, Department of Medicine, Communicable Disease Center, Hamad Medical Corporation, Doha, Qatar; 6 Jordan Hospital, Amman, Jordan; 7 Ibn Sina University of Medical and Pharmaceutical Sciences, Baghdad, Iraq; 8 Department of Pediatrics, College of Medicine and Health Sciences, United Arab Emirates University, Al Ain, UAE; 9 Pakistan Kidney and Liver Institute and Research Center, Lahore, Pakistan; 10 Clinic for Infectious Diseases, Sarajevo, Bosnia and Herzegovina; 11 University Medical Centre Ljubljana, Ljubljana, Slovenia; 12 Dr. Fran Mihaljevic University Hospital for Infectious Diseases, University of Zagreb School of Medicine, HR Zagreb, Croatia; 13 Department of Infectious and Tropical Diseases, Asfendiyarov Kazakh National Medical University, Almaty, Kazakhstan; 14 Center for Infectious Diseases, Baku, Azerbaijan; 15 Hospital for Infectious Diseases, Skopje, Republic of North Macedonia; 16 Faculty of Medicine, Infectious Disease Department, Tirana, Albania; 17 Infectious Diseases Clinical Hospital Toma Ciorba, Chisinau, Moldova; 18 American University of Beirut Medical Center, Beirut, Lebanon; 19 Hospital Sungai Buloh, Sungai Buloh, Selengor Malaysia; 20 International Higher School of Medicine, Bishkek, Kyrgyz Republic; 21 Loghman Hakim Hospital, Tehran, Iran; 22 King Salman Humanitarian Aid and Relief Center & College of Medicine, Alfaisal University, Riyadh, Saudi Arabia. Hubert Department of Global Health, Rollins School of Public Health, Emory University, Atlanta, USA; 23 Infectious Diseases Sf. ParaschevaIasi, Iasi, Romania; 24 Pinea Medical Center, Prishtina, Kosovo; 25 Department of Infectious Diseases and Clinical Microbiology, Aydın Adnan Menderes University Faculty of Medicine, Aydın, Turkey; 26 Faculty of Medicine, Department of Medical Microbiology and Immunology “Prof. Elissay Yanev”, Medical University of Plovdiv, Plovdiv, Bulgaria; 27 President of Serbian ID Society, Belgrade, Serbia; 28 Central Research Institute of Epidemiology, Moscow, Russia; 29 Kandahar University Teaching Hospital, Kandahar, Afghanistan; 30 Bukhara Medical University after named Abu Ali ibn Sino, Bukhara, Uzbekistan; 31 Department of Infectious Diseases and Clinical Microbiology, Çukurova University Faculty of Medicine, Adana, Turkey; 32 Attikon University Hospital, Athens, Greece; 33 Christian Medical Hospital, Vellore, India

**Keywords:** Cross-sectional studies, Health workforce, Infectious diseases, Medical education, Specialty training

## Abstract

**Purpose:**

Increasing travel, climate change, spread of antimicrobial resistance and pandemics increased the need for well-trained infectious diseases (ID) specialists and qualified ID specialist training for protecting public health all over the world. In this study, we aimed to provide a comprehensive overview of ID specialty training programs for standardization and quality improvement in a large geographical area.

**Methods:**

We conducted a cross-sectional study among national respondents of 29 countries [Central Asia (Azerbaijan, Uzbekistan, the Kyrgyz Republic, Kazakhstan), the Middle East (Iran, Saudi Arabia, Jordan, Iraq, Oman, the United Arab Emirates, Qatar, Lebanon), Southeast Europe (Albania, Greece, Kosovo, Slovenia, Bosnia and Herzegovina, Serbia, the Republic of North Macedonia, Croatia), Eastern Europe (Russia, Moldova, Romania, Bulgaria), South Asia (India, Pakistan, Afghanistan), Southeast Asia (Malaysia), Türkiye] to evaluate the structure and components of ID training programs.

**Results:**

In this study, structural variability in ID training programs was notable. 65.5% of the countries offered independent specialty program, 59% of the countries reported a required exam for entry into the ID specialization. Nearly all of the countries had a formal training curriculum; written exams were the most common used assessment method.

**Conclusion:**

This study provides a comprehensive overview of ID specialty training across diverse regions, highlighting major structural differences in curricula, training duration, and national standards. Its broad geographic scope and contributions from actively engaged ID educators offer a unique global perspective. The findings underscore the urgent need for harmonized training frameworks, the strengthening of national curricula, and the promotion of international collaboration and inclusive strategies, all essential for developing a skilled, competent and resilient global ID workforce.

**Supplementary Information:**

The online version contains supplementary material available at 10.1007/s15010-025-02597-7.

## Introduction

The specialty of infectious diseases (ID) remains a cornerstone of global public health requiring specialized medical training, expertise and robust healthcare systems to manage all kind of infectious diseases, including emerging infections, potential pandemics and antimicrobial resistance. The Infectious Diseases Society of America (IDSA) and the European Society of Clinical Microbiology and Infectious Diseases (ESCMID) define ID specialists as physicians with advanced training in the diagnosis, treatment, prevention and consultation of infectious diseases. Accordingly, ID specialists play a critical role in managing complex infections, antibiotic-resistant pathogens, emerging infectious threats and global pandemics in both the hospitals and the outpatient settings. The recognition of ID as a distinct specialty and the harmonization of specialist training are essential for strengthening healthcare resilience and response capacity [1, 2].

ID training equips physicians with expertise in clinical ID management, epidemiology, public health and infection control [3]. However, the structure, curriculum, duration and delivery of ID specialty training programs vary significantly between countries, influenced by healthcare systems, geographic varieties, available resources and social needs. In Europe, efforts have been made to standardize ID specialty training through guidelines provided by the European Union of Medical Specialists (UEMS) and ESCMID. These organizations have outlined core competencies, curriculum requirements and evaluation methods to harmonize training across the region. Despite these efforts, notable heterogeneity still persists within European countries, while some countries fully complying with ESCMID standards, others modify their programs according to local needs [4, 5].

Studies on ID specialty training were mostly focused on high-income countries or associated with UEMS and ESCMID, there were only a few publications evaluating the structure of ID training programs across low and middle-income countries.For this reason we aimed to provide a comprehensive overview of ID specialty training programs worldwide, focusing on key components such as application and acceptance process, related subspecialties, curriculum, duration, clinical rotations and competencies. It also seeks to identify the strengths, gaps and opportunities for standardization and quality improvement in ID specialty training globally. By including data from 29 countries across Europe, Asia, and the Middle East, this study offers to be one of the first cross-regional comparison of ID specialty training. Unlike prior European-centric analyses, it provides original insights into structural differences, training challenges, and evolving perceptions of the specialty in the post-COVID era. By generating expert-informed, country-level data across diverse settings, this study contributes novel evidence that can guide efforts toward more equitable, competency-based, and internationally aligned ID training models.

## Methodology

### Study design and participants

**Fig. 1:**
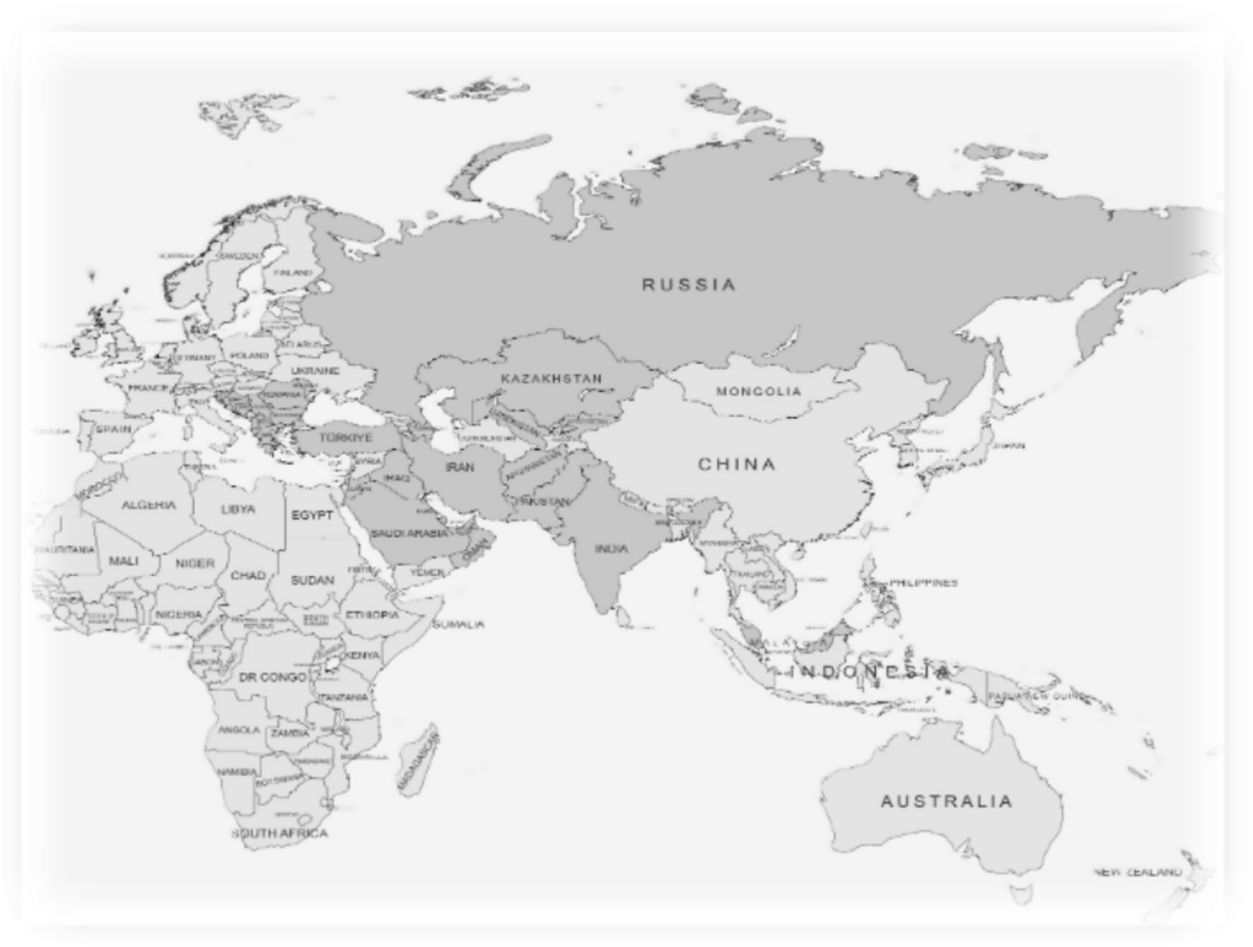
Countries included in the study are marked in dark grey color [Generated using MapChart.net]

This study utilized a descriptive, cross-sectional design. An initial invitation to participate in the study was distributed through the Infectious Diseases International Research Initiative (ID-IRI) network, a voluntary academic community of ID specialists and trainers. A detailed information letter accompanied the invitation, clearly outlining the aim, scope, and voluntary nature of the study. The letter emphasized that the survey sought structured, national-level data on ID specialty training, not personal opinions or institutional data, and that participation was anonymous and voluntary. Respondents, were instructed to complete the questionnaire independently and encouraged to consult national guidelines or authorities if needed. A total of 29 scholars from 29 different countries, qualified professionals actively involved in ID specialty training programs, participated in the study and provided national-level insights. Although only one respondent per country was included, which may limit generalizability within each country, this approach ensured consistency and allowed for systematic cross-country comparison of training structures and policies. The participants represented five geographic regions: Central Asia (Azerbaijan, Uzbekistan, Kyrgyz Republic, and Kazakhstan), the Middle East (Iran, Saudi Arabia, Jordan, Iraq, Oman, United Arab Emirates, Qatar, and Lebanon), the Southeast Europe (Albania, Greece, Kosovo, Slovenia, Bosnia and Herzegovina, Serbia, Republic of North Macedonia, and Croatia), Eastern Europe (Russia, Moldova, Romania, and Bulgaria), South Asia (India and Pakistan, Afghanistan), Southeast Asia (Malaysia), and Türkiye (Fig. 1).

This study did not involve patients, medical records, clinical interventions, or any sensitive personal data. It surveyed ID specialists or trainers about the structure and policies of postgraduate ID training in their respective countries. All information provided by participants was based on publicly known or professionally accessible training structures, and no institutional or confidential data were used. The data collection tool was developed specifically for this study. Participation was fully voluntary and anonymous, and no incentives were offered. Informed consent was implied through participants' voluntary completion of the online questionnaire.

### Data collection and analysis

A structured data collection tool (Supplement 1) was developed by a professional team comprising one medical education specialist and three ID specialists. The tool was informed by relevant references and existing literature to ensure content validity and comprehensiveness. It was pilot-tested with three independent ID specialists and reviewed by two medical education experts. Feedback from this process was incorporated to enhance clarity and usability. The final questionnaire consisted of three main sections:

Part 1 – Professional Background: Focused on general professional information, such as academic title, country of practice, and primary specialty. No personally identifiable or sensitive data were collected.

Part 2 – General Structure of ID Practice and Training: Explored the national structure and current status of ID specialty training, using 19 targeted questions (both multiple choice and open-ended) designed to assess the scope, organization, and challenges of ID practice in each country.

Part 3 – ID Specialty Training Curriculum: Assessed the specific components of ID specialty curricula through 10 multiple choice and open-ended questions, addressing duration, comprehensiveness, and educational content.

A total of 29 participants from 29 countries completed the questionnaire independently via Google Forms between 15 October and 30 November 2024. Two reminder emails were sent during the data collection period to encourage full participation. In cases of incomplete or unclear responses, participants were encouraged to consult relevant authorities or provide explanatory comments. Although only one qualified respondent per country contributed, limiting generalizability within each nation, this approach ensured consistency and enabled systematic cross-country comparison.

Descriptive statistics were used to summarize all survey responses. To explore regional differences in categorical variables, whether ID is recognized as an independent specialty or whether a thesis is required, Fisher’s exact test was applied due to small expected frequencies in some cells. For continuous variables, such as training duration, number of training centers, and number of trainees, one-way ANOVA was used to assess variation across regions, based on the United Nations regional classifications. A p-value of < 0.05 was considered statistically significant.

### Results

The study included participants from 29 countries, representing a diverse range of medical professionals specializing in ID and related fields. Of the participants, 20 specialized in infectious diseases, 2 in both infectious diseases and Clinical Microbiology, 2 in Clinical Microbiology, 4 in both infectious diseases and internal medicine and 1 in infectious diseases and Pediatrics. The participants represented a variety of academic titles, such as MDs, MScs, PhDs, Assistant Professors, Associate Professors and Full Professors.

#### Variations in ID specialty configuration across different countries

Participants from nineteen (65.5%) countries indicated that ID is recognized as an independent specialty program that can be pursued directly after medical school in their country. In countries such as Greece, Jordan, Oman, Qatar, Pakistan, India, Lebanon and Malaysia, ID is pursued as a specialty following the completion of Internal Medicine. In the United Arab Emirates and Saudi Arabia, ID is a specialty pursued after completing internal medicine or pediatrics (Table 1). No statistically significant association was found between geographic region and the organization of ID as an independent specialty (Fisher’s exact test, p = 0.545).

**Table 1:** General structure of ID specialty training programs

Country	Mandatory national written exam for ID specialty entry (exam name)	Status of ID Specialty	Total ID specialists (national level)	Total ID training centers (national level)	ID training duration (years)	Opportunity for training abroad	Licensing exam frequency & renewal period	Academic career pathway for ID specialists (PhD requirement)
Afghanistan	Exam by national authorities—**Specialty Program Entrance Exam**	**Independent specialty**	NA	4	4	–	No	No PhD is required
Albania	Each institution makes its own	**Independent specialty**	60	1	4	–	No	PhD is required
Azerbaijan	Exam by national authorities—**Residency Entrance Exam**	**Independent specialty**	NA	7	4	–	**Yes, 5y**	PhD is required
Bosnia and Herzegovina	No written exam is needed	**Independent specialty**	120	4	5	–	No	PhD is required
Bulgaria	Each institution makes its own	**Independent specialty**	40	5	4	–	No	No PhD is required
Croatia	Final specialist examination in ID*	**Independent specialty**	150	4 regional including 1 national	5	** + **	**Yes, 6y**	PhD is required
Greece	No written exam is needed	Specialty following IM	253	12	2	–	No	PhD is required
India	Exam by national authorities—**Super Specialty NEET**	Specialty following IM	200	13	3	–	No	No PhD is required
Iran	Exam by national authorities—**National Iranian Residency Program**	**Independent specialty**	1200	22	4	–	**Yes, 5y**	No PhD is required
Iraq	Exam by local authorities	**Independent specialty**	20–30	2	5	–	No	No PhD is required
Jordan	Exam by national authorities—**Jordan Medical Council**	Specialty following IM	10	0	2	–	No	No PhD is required
Kazakhstan	Exam by national authorities – **Final State Certification**	**Independent specialty**	NA	7	3	–	**Yes, 5y**	No PhD is required
Kosovo	No written exam is needed	**Independent specialty**	36	1	4	–	No	PhD is required
Kyrgyz Republic	Each institution makes its own	**Independent specialty**	240	3	3	-	**Yes, 5y**	No PhD is required
Lebanon	No written exam is needed	Specialty following IM	110	7	2	–	No	No PhD is required
Malaysia	No written exam is needed	Specialty following IM	66	16	2	–	No	No PhD is required
Moldova	Nı written exam is needed	Independent specialty	160	2	4	–	**Yes, 5y**	PhD is required
North Macedonia	No written exam is needed	**Independent specialty**	51	2	5	–	**Yes, 7y**	PhD is required
Oman	No written exam is needed (Written exam is needed for IM)	Specialty following IM	15	2	4	** + **	No	No PhD is required
Pakistan	No written exam is needed	Specialty following IM	50	9	2	–	No	No PhD is required
Qatar	Exam by national authorities—**IDSA**	Specialty following IM	33	1	3	–	No	No PhD is required
Romania	Exam by national authorities—**Residency Exam**	**Independent specialty**	1427	11	5	–	No	No PhD is required
Russia	Exam by national authorities	**Independent specialty**	7230	81	2	–	**Yes, 5y**	PhD is required
Saudi Arabia	Exam by national authorities—**Saudi Board**	Specialty following IM or pediatrics	> 100	> 30	3	** + **	No	No PhD is required
Serbia	No written exam is needed	**Independent specialty**	160	5	3	–	**Yes, 7y**	PhD is required
Slovenia	No written exam is needed	**Independent specialty**	73	5	6	–	No	PhD is required
Türkiye	Exam by national authorities—**Medical Specialization Exam**	**Independent specialty**	> 1200	> 80	5	–	No	No PhD is required
United Arab Emirates	Exam by national authorities **-Emirati Board (National Institute for Health Specialties)**	Specialty following IM or pediatrics	NA	2	2	–	No	No PhD is required
Uzbekistan	Each institution conducts itself	**Independent specialty**	NA	NA	2	–	No	No PhD is required

*In Crotia final specialist examination in ID is mandatory before an Examination Committee composed of three members (ID specialist & University Professors), consisting of a theoretical and practical part, taken in the institution where the president of the Examination Committee is employed, mostly a professor from the Department of ID at the leading national University Hospital for ID

#### Pathways to ID specialty

Participants from seventeen (59%) countries reported a required exam for entry into the ID specialization, administered by national authorities (n = 13) or local authorities (n = 4). A written exam was not required in 11 (34%) countries, in Oman written exam requirement was reported for IM specialization prior to ID. The names of the entrance exams for each country are listed in Table 1. In Russia, the exam is conducted by national authorities as a primary accreditation after graduation from medical school (with scores), which serves as the admission examination for residency in ID and other specialties (Table 1).

#### Occupancy rates for ID training positions

Participants were asked to indicate the occupancy rate (%) of the available training positions for ID in the most recent specialty exams in their country. Professionals from Greece, Azerbaijan, Oman, Qatar, Romania, Iraq, Moldova, Afghanistan and Uzbekistan reported a 100% occupancy rate for available ID training positions in the most recent specialty exams in their countries. Russia similarly reported 95–100% occupancy rate. Croatia and the Kyrgyz Republic reported a 90% occupancy rate, while 15 other countries reported an occupancy rate over 80%. Five professionals were unable to provide any data (Table 1).

#### Perception of ID specialty in a post-COVID era

The general expression of ID specialty in the country following the COVID pandemic was rated on a scale of 1 to 5 (1 = no perceived increase in importance, 3 = moderate change, 5 = clearly perceived increase in importance), with scores ranging from 2 to 5 (mean 3.5). The lowest rating was reported in Slovenia (1), while the highest ratings were in Iraq, Malaysia, and Uzbekistan (5).

#### Variations in ID specialists and training centers across the countries

The number of reported ID specialists and training centers varied significantly among the countries. The number of ID specialists ranged from 10 to 7230. Jordan did not report any training centers. While countries such as Kosovo and Albania reported only one and the UAE reported two training centers, Türkiye reported over 80; and Russia reported 81 training centers (Table 1).

#### Length of ID specialty training

The duration of ID specialty training varied, with an average of 4 years (range 1–6 years). Seven countries reported a training duration of 1–2 years, 13 countries had duration with in the 3–4 years range, and seven countries reported duration of 5 or more years. The longest duration, 6 years, was reported from Slovenia (Table 1). A one-way ANOVA showed a statistically significant difference in ID training durations across regions (F (5,23) = 9.40, p < 0.001).

#### Compulsory training in other institutions

Compulsory period of training in other local centers or abroad included in the ID specialty training program was positive in Kosovo, Oman, Croatia, Saudi Arabia and Kazakhstan. In Kosovo trainees are offered free training periods conducted in other institutions or abroad for ID specialty training curriculum or financed by the individuals themselves. In Oman, ID specialty trainees need to complete a fellowship abroad for at least 2 years. In Croatia, part of the rotations should be performed only at university hospitals (4 regional, including one national university center) in addition to rotations performed in general hospitals, depending on the authorization to conduct specialist training held by an individual health institution, depending on meeting general and special conditions (organization, equipment, number of specific examinations and procedures, number of patients, number of university staff, etc.) prescribed by the rulebook and regulations for each specialization issued by the Ministry of Health. In Saudi Arabia, trainees rotate through various hospitals and may spend one year in Canada, depending on their specialization location and in Kazakhstan a preferred academic program was reported, not mandatory (Table 1).

#### License renewal

In the study, nine countries reported to have mandatory periodic license exam for ID. The most common renewal period of the exam was every 5 years, in six countries: Azerbaijan, Iran, Russia, Kazakhstan, Kyrgyz Republic, and Moldova. A few exceptions had longer renewal periods, as Croatia (6 years), Serbia and North Macedonia (7 years) (Table 1).

#### Postgraduate academic career pathways

In the majority of the countries (n = 19; 66%) ID specialists may continue their academic career after graduation without obtaining a PhD. However, in some countries (n = 10; 34%) a PhD was mandatory for academic progression. The countries requiring a PhD for academic advancement were Jordan, Azerbaijan, Kosovo, Qatar, Romania, India, Iran, North Macedonia and Malaysia (Table 1).

#### ID training curriculum

Nearly all of the countries (n = 27; 93%) reported having a formal ID specialty training curriculum, with the exception of Oman and Serbia. Twenty-four countries (83%) reported having national ID associations in the participating countries. The names and web sites of these associations are listed in Supplement 2.

#### ID subspecialties

Ten (35%) countries (Bosnia and Herzegovina, Croatia, Iran, Kyrgyz Republic, Oman, Qatar, Russia, Serbia, Türkiye and Uzbekistan) reported that they have some subspecialties under the ID specialty: Intensive Care (n = 6, 21%), Parasitology (n = 4, 14%), HIV&AIDS (n = 4, 14%), Immunology & Allergy (n = 4, 14%), Epidemiology (n = 4, 14%), Microbiology (n = 3, 10%), Hepatology (n = 3, 10%) and Virology (n = 2, 7%). In Türkiye, Immunology & Allergy is replaced with Basic Immunology (Table 2).

**Table 2:** Subspecialties of ID training programs

Country	Intensive Care	Parasitology	HIV&AIDS	Immunology & Allergy	Epidemiology	Microbiology	Virology	IPC	Hepatology	Public health	Travel medicine	Immunocompromised host	Clinical pharmacology	Geriatrics	Tuberculosis	Leprosy	STDs
Bosnia and Herzegovina	✓			✓	✓				✓								
Croatia	✓			✓													
Iran			✓					✓				✓					
Kyrgyz Republic		✓	✓														
Russia	✓	✓	✓	✓	✓	✓				✓							
Oman	✓					✓					✓						
Qatar		✓	✓		✓	✓	✓		✓						✓	✓	✓
Serbia	✓				✓		✓						✓	✓			
Türkiye	✓			✓													
Uzbekistan		✓															

*IPC* infection prevention and control, *STDs* sexually transmitted diseases

#### Curriculum topics

The curriculum topics related to rational antibiotic use; healthcare-associated infections and critical care/intensive care, and travel medicine were among the most prevalent across the countries. The specialized topic of health economics appeared to be included only in Jordan, while palliative care was included only in Slovenia (Table 3).

**Table 3:** Distribution of topics in the curriculum

Country	Rational antibiotic use	Healthcare associated infections	IPC	Critical intensive care	Research methodology	Travel medicine	Clinical epidemiology	Immunization	Team work	Wound care	Immunology	Laboratory management	Communication skills	Leadership	Quality improvement	Palliative care
Albania	✓	✓	✓	✓	✓	✓	✓	✓	✓	✓	✓		✓	✓	✓	
Azerbaijan	✓	✓			✓		✓				✓					
Bosnia & Herzegovina	✓	✓	✓	✓	✓	✓	✓	✓	✓		✓		✓	✓	✓	
Bulgaria	✓	✓	✓	✓	✓	✓		✓	✓		✓		✓	✓	✓	
Croatia	✓	✓	✓	✓	✓	✓	✓	✓	✓	✓	✓	✓	✓		✓	
Greece	✓	✓	✓	✓		✓		✓								
India	✓	✓	✓	✓	✓	✓	✓	✓	✓		✓		✓		✓	
Iran	✓	✓	✓	✓	✓	✓		✓	✓				✓		✓	
Iraq	✓	✓	✓	✓	✓	✓		✓	✓				✓		✓	
Jordan	✓	✓	✓	✓	✓	✓	✓	✓	✓	✓	✓	✓	✓	✓		
Kazakhstan	✓	✓	✓	✓	✓	✓	✓	✓	✓	✓	✓	✓	✓	✓	✓	
Kosovo	✓	✓	✓	✓	✓	✓	✓	✓	✓	✓	✓	✓	✓	✓		
Kyrgyz Republic	✓	✓	✓	✓	✓	✓	✓	✓	✓		✓	✓	✓	✓		
Lebanon	✓	✓	✓	✓	✓		✓	✓	✓				✓	✓		
Malaysia	✓	✓	✓	✓	✓	✓	✓	✓	✓		✓		✓	✓		
Moldova	✓	✓	✓	✓	✓	✓		✓	✓				✓		✓	
North Macedonia	✓	✓	✓	✓	✓	✓	✓	✓	✓				✓	✓		
Pakistan	✓	✓	✓	✓	✓	✓	✓	✓	✓	✓	✓	✓	✓	✓	✓	
Qatar	✓	✓	✓	✓	✓	✓	✓	✓	✓				✓	✓		
Romania	✓	✓	✓	✓	✓	✓	✓	✓	✓				✓	✓	✓	
Russia	✓	✓	✓	✓	✓	✓	✓	✓	✓				✓	✓	✓	
Saudi Arabia	✓	✓	✓	✓	✓	✓	✓	✓	✓				✓	✓	✓	
Serbia	✓	✓	✓	✓	✓	✓	✓	✓	✓				✓	✓	✓	
Slovenia	✓	✓	✓	✓	✓	✓	✓	✓	✓	✓	✓	✓	✓	✓	✓	✓
Türkiye	✓	✓	✓	✓	✓	✓	✓	✓	✓			✓	✓	✓	✓	
UAE	✓	✓	✓	✓	✓	✓	✓	✓	✓				✓	✓	✓	
Uzbekistan	✓	✓	✓	✓	✓	✓	✓	✓	✓				✓	✓	✓	

*IPC* infection prevention and control

#### Invasive procedural competencies

Invasive procedures that the ID trainees are required to be proficient were listed as; lumbar puncture (n = 23, 79%), urinary catheter insertion (n = 16, 55%), pleural fluid drainage (n = 12, 41%), abscess drainage (n = 6, 21%), endoscopic intervention (n = 3, 10%), bone marrow aspiration (n = 2, 7%), bone marrow biopsy (n = 1, 3%), joint aspiration (n = 1, 3%), central venous access (n = 1, 3%), skin biopsies (n = 1, 3%), endotracheal intubation (n = 1, 3%) and abdominal paracentesis (n = 1, 3%) (for trainees working in the Intensive Care Unit).

#### Mandatory rotations

The ID specialty training programs across countries encompass a variety of integral components and mandatory rotations. Internal medicine and microbiology were reported as core components of all programs, followed by infection control, epidemiology, and antimicrobial stewardship (AMS) as essential rotations. Sexually transmitted diseases, travel medicine, and transplantation were included in selected programs. Rotations in Pediatrics, Radiology, Neurology, Hepatology, and Intensive Care were integrated into some curricula. The duration of rotations varies significantly, ranging from 2 weeks to 4 years (Table 4).

**Table 4:** Integral parts/rotations of ID Specialty Training Program

Country	internal medicine	Microbiology	IPC	STDs	Travel medicine	Transplantation	Immunization	Epidemiology	Pediatrics	Radiology	Neurology	Hepatology	Intensive Care	Antimicrobial Stewardship	Others	Duration of the rotations
Afghanistan	✓	✓			✓							✓			Hematology, Parasitology Central Lab	1–10 months
Albania	✓	✓	✓	✓	✓	✓	✓	✓	✓	✓	✓	✓			Pneumology, Nephrology, Cardiology, Gastro ENT	3–4 weeks each
Azerbaijan	✓	✓					✓		✓						Pathology, Pulmonology, Allergy	51 weeks total
Bosnia & Herzegovina	✓	✓	✓		✓		✓									16 months
Bulgaria	✓	✓					✓									–
Croatia	✓	✓	✓	✓	✓	✓	✓		✓	✓	✓	✓	✓	✓	Cardiology, Gastroenterology, Endocrinology, Pulmonology, Nephrology, Haematology, Immunology, Rheumathology, Oncology, Pharmacology	3 years ID + 2 years in IM common trunk
Greece	✓		✓		✓		✓	✓								2 months
India	✓	✓	✓	✓		✓		✓								3 years
Iran	✓	✓	✓		✓		✓								ID in immunocompromised host	1.5 years
Iraq	✓	✓	✓	✓	✓		✓		✓							2 years IM, then 3 years ID rotations
Jordan	✓	✓	✓	✓	✓	✓	✓									2 years
Kazakhstan	✓			✓			✓								Children ID in hospital, ID in outpatient clinic, Elective disciplines	–
Kosovo	✓	✓	✓				✓	✓	✓	✓	✓	✓	✓		Pulmonology, Dermatovenerology, Cardiology, Nephrology, Hematology, Gastroenterology	4 years
Kyrgyz Republic	✓	✓			✓		✓								Surgery, Obstetrics and Gynecology, Internal Medicine, Pediatrics, Emergency Care, ID in outpatient Department	10 to 12 weeks in first year
Lebanon		✓	✓	✓	✓	✓	✓									1 month
Malaysia	✓	✓	✓			✓		✓								1–3 months
Moldova	✓	✓		✓			✓						✓		Pediatrics, Clinical Pharmacology	2–8 weeks
North Macedonia	✓	✓			✓		✓		✓				✓		Dermatology, Gastroenterology, Nephrology, Cardiology, Hematology & Oncology, Endocrinology, Toxicology, Rheumatology, Pulmonology	1 month
Oman	✓	✓	✓	✓			✓	✓								2 years
Pakistan		✓	✓			✓									Tuberculosis & HIV training centers	4–6 months
Qatar	✓	✓	✓	✓	✓	✓		✓						✓		13 blocks per year
Romania	✓	✓		✓			✓	✓	✓		✓		✓		Emergency Medicine, Nephrology, Diabetes & Nutrition	3 months-4 months each
Russia	✓		✓		✓		✓	✓							Public Health	3 months
Saudi Arabia		✓	✓				✓	✓							–	6 weeks
Serbia	✓	✓					✓	✓	✓						Dermatovenerology, Emergency Medicine	3 months
Slovenia	✓	✓	✓	✓	✓									✓	Immunocompromised, Palliative Care	6 weeks
Türkiye	✓	✓	✓	✓	✓	✓	✓	✓	✓	✓	✓		✓	✓	Pulmonology	GIM (6 months), IC (2 months), Pulmonology (1 month), Radiology (1 month)
UAE	✓	✓	✓	✓	✓	✓	✓	✓							–	4 weeks block
Uzbekistan			✓	✓			✓								–	3 years

*IPC* infection prevention and control, *STDs* sexually transmitted diseases

#### Educational methods

Although traditional methods such as lectures, didactic sessions, practical exercises, printed materials, manuals, and hands-on workshops were adopted by all countries, educational methods were reported to vary across them. Technology-enhanced methods, including webinars, virtual conferences, and the use of printed materials/manuals, were frequently utilized. Mobile apps, role-playing and scenario-based training were the least adopted methods, appearing only in a few countries, such as Croatia, Russia, Türkiye and Kazakhstan (Table 5).

**Table 5:** Educational methods in ID training programs

Country	Traditional (Lectures, didactic sessions)	Practical exercises	Online & e-learning activities	Webinars & virtual conferences	Field training (observation, community-based)	Hands on workshops (simulation practices)	Printed materials & manuals	Mobile apps	Journal clubs	Study groups	Role playing, scenario-based training
Afghanistan	✓	✓		✓	✓	✓	✓				
Albania	✓	✓	✓	✓		✓	✓	✓			
Azerbaijan	✓	✓			✓	✓	✓				
Bosnia and Herzegovina	✓	✓	✓	✓		✓	✓	✓	✓	✓	✓
Bulgaria	✓	✓			✓	✓	✓				
Croatia	✓	✓	✓	✓	✓	✓	✓	✓	✓	✓	✓
Greece	✓	✓	✓	✓		✓	✓	✓	✓	✓	✓
India	✓	✓			✓	✓	✓				
Iran	✓	✓	✓	✓	✓	✓	✓	✓	✓	✓	✓
Iraq	✓	✓	✓	✓		✓	✓	✓	✓	✓	✓
Jordan	✓	✓	✓	✓	✓	✓	✓	✓	✓	✓	✓
Kazakhstan	✓	✓				✓	✓				
Kosovo	✓	✓	✓	✓		✓	✓		✓	✓	✓
Kyrgyz Republic	✓	✓	✓	✓		✓	✓	✓	✓	✓	✓
Lebanon	✓	✓	✓	✓	✓	✓	✓	✓	✓	✓	✓
Malaysia	✓	✓	✓	✓	✓	✓	✓	✓	✓	✓	✓
Moldova	✓	✓	✓	✓		✓	✓	✓	✓	✓	✓
North Macedonia	✓	✓	✓	✓		✓	✓	✓	✓	✓	✓
Oman	✓	✓			✓	✓	✓				
Pakistan	✓	✓	✓	✓	✓	✓	✓	✓	✓	✓	✓
Qatar	✓	✓	✓	✓		✓	✓	✓	✓	✓	✓
Romania	✓	✓	✓	✓		✓	✓	✓	✓	✓	✓
Russia	✓	✓	✓	✓	✓	✓	✓	✓	✓	✓	✓
Saudi Arabia	✓	✓	✓	✓	✓	✓	✓	✓	✓	✓	✓
Serbia	✓	✓	✓	✓	✓	✓	✓	✓	✓	✓	✓
Slovenia	✓	✓	✓	✓		✓	✓	✓	✓	✓	✓
Türkiye	✓	✓	✓	✓		✓	✓	✓	✓	✓	✓
UAE	✓	✓	✓	✓		✓	✓	✓	✓	✓	✓
Uzbekistan	✓	✓	✓	✓		✓	✓	✓	✓	✓	✓

#### Assessment methods

Assessments of ID trainees are listed in Table 6. The logbook/portfolio (n = 19; 66%) was the most commonly used assessment method, followed by the annual progress/professional development exam (n = 16; 55%). The most frequently reported summative formal exams were written exams (n = 17; 59%), oral exams (n = 14; 48%), and clinical exams (n = 13; 45%). The objective structured clinical examination (OSCE) was the least frequently used method, appearing in only 2 countries. The thesis requirement, referring to the mandatory submission of a research-based or project-based document as part of the criteria for completing ID specialty training, was mandated by only 7 countries (24%) in the dataset (Table 6).

**Table 6:** Assessment of ID trainees

Country	Logbook/Portfolio	Annual exam	Summative formal exam					Thesis
			Written	Oral	Clinical	Lab	OSCE	
Afghanistan		✓	✓		✓			
Albania		✓	✓					✓
Azerbaijan		✓	✓					
Bosnia and Herzegovina	✓	✓						
Bulgaria								
Croatia	✓			✓	✓	✓		
Greece	✓		✓	✓				
India			✓	✓	✓			
Iran	✓	✓	✓		✓		✓	✓
Iraq	✓		✓	✓	✓			
Jordan			✓				✓	✓
Kazakhstan	✓		✓					
Kosovo	✓	✓		✓	✓	✓		
Kyrgyz Republic	✓	✓	✓	✓	✓			
Lebanon		✓						
Malaysia	✓	✓		✓				
Moldova	✓	✓	✓	✓	✓			
North Macedonia	✓	✓		✓	✓			✓
Oman								
Pakistan	✓		✓		✓			
Qatar	✓							
Romania	✓		✓		✓	✓		✓
Russia	✓	✓	✓	✓				
Saudi Arabia		✓	✓					
Serbia				✓	✓			
Slovenia	✓			✓	✓			
Türkiye	✓	✓	✓	✓				✓
United Arab Emirates	✓	✓						
Uzbekistan	✓	✓	✓	✓				✓

Fisher’s exact test was used to examine associations between categorical variables such as specialty independence (p = 0.545), thesis requirement (p = 1.000), and OSCE implementation (p = 1.000) across selected geographic regions. No statistically significant associations were observed.

## Discussion

ID specialists play an essential role in managing infectious diseases and public health challenges worldwide [6]. This study presents a comprehensive analysis of ID specialty training in 29 countries, highlighting significant variability in training programs across them. The findings align with existing literature, including European efforts by ESCMID and UEMS to standardize ID training and global studies assessing the evolution of ID training [5, 7].

One notable finding from our study was the structural variability in ID training programs. While 65.5% of the countries offered ID as an independent specialty, others required prior IM or Pediatrics training. Although this aligns with UEMS data, showing 75% of European countries do the same, there is a need for global consensus on ID training structures [2, 8]. Some studies suggest that independent ID training may be more effective, especially in regions with high infectious disease burdens, as the shorter pathway to specialization could encourage more candidates to pursue the field [9, 10]. Conversely, others suggest prior IM training may enhance clinical reasoning skills and the support diagnostic accuracy [7, 11].

In this study, entry requirements also varied significantly; 58.6% of the countries reported mandatory entrance exams conducted either at the national or institutional level. These exams are primarily intended to assess the academic preparation, clinical skills, problem-solving, decision-making and critical thinking abilities of ID fellowship candidates. Structured selection processes may lead to more consistently trained specialists; however, some studies suggest that they may redirect attention from clinical practice to test preparation, potentially misaligning with the realities of medical care [12, 13]. Russia's entrance system, like Australia's CV-based model, emphasizes comprehensive evaluation over a single exam, considering accreditation scores, research, clinical experience and interviews [14]. In several Balkan countries where ID follows IM, selection is based on institutional evaluations or clinical experience rather than national exams. Although national exams may not suit every context, the absence of structured assessment can lead to differences in training quality. Common methods like exams, interviews or portfolios can help ensure quality while respecting institutional autonomy. Regional or global collaboration may further support recognition and workforce planning. On the other hand, only approximately one-third of the countries implemented a system of periodic license renewal following the completion of specialization in infectious diseases, indicating a limited emphasis on ongoing professional validation and continued competency assessment within the specialty time [5].

ID specialty training durations varied across countries, with a mean of 4 years and a range of 1–6 years. Approximately one-third of the countries in this study offered formal subspecialties within ID, including Intensive Care, HIV/AIDS, Parasitology, Immunology and Microbiology. All ID programs included internal medicine and microbiology rotations, while infection control, epidemiology and AMS were present in most curricula; aligning with ESCMID recommendations [5]. However, there was a significant variability in the structure and duration of rotations (from 3 weeks to 3 years) in our study. Accordingly, some countries reported to have longer and more diverse rotations, while others have limited training periods [4, 15]. In our study IM and Microbiology were the core components of ID training programs across the countries.

In addition to training formats and mobility, the content of ID curricula reveals shared priorities and notable gaps. Core topics included rational antibiotic use (100%), healthcare-associated infections (100%), intensive care (97%), infection prevention and control (IPC) (97%), immunization (97%), and travel medicine (97%). Particular areas such as internal medicine, microbiology, epidemiology and AMS appear essential and should form the basis of a standardized global ID curriculum. The most important interdisciplinary areas in which ID physicians play important roles are AMS and IPC. A study by Maraolo and al. showed that only 32% of European countries had both guidance and national requirements regarding AMS programs, in contrast to 61% for IPC. Formal national staffing standards for AMS and IPC hospital-based activities were present in 24 and 63% of European countries, respectively emphasizing that organization and training of AMS and IPC in Europe are heterogeneous and national requirements for activities are frequently missing as well as harmonization at the international level [16].

ID specialty training programs differ in how much they emphasize procedural competency. The study found that lumbar puncture was the most commonly reported invasive procedure, urinary catheter insertions and pleural fluid drainage were also frequent. These findings align with the previous research indicating variability in procedural exposure based on regional training priorities [17].

Regional differences in ID patterns and increasing global travel require broader clinical exposure in training. International rotations can strengthen preparedness by familiarizing trainees with diverse health systems. In our study, participants took part in mandatory or voluntary rotations in Kosovo, Oman, Saudi Arabia and Kazakhstan, reflecting European cross-border training efforts to foster global competency [7].

Alongside differences in content and rotations, this study also revealed notable differences in the educational methods employed across ID training programs. While traditional methods, such as lectures, printed materials and hands-on workshops, remain widely used, many countries have begun incorporating modern, technology-enhanced formats including webinars and virtual conferences, reflecting an on-going trend toward more flexible learning environments. However, the adoption of interactive and learner-centered methods, such as simulation-based training, mobile applications, role-playing and scenario-based learning remains limited. These approaches, which are essential for developing clinical reasoning, decision-making and real-world preparedness, were reported only in a few countries, including Croatia, Russia, Türkiye, and Kazakhstan. As competency-based training becomes more important, there is a clear need to adopt more structured, interactive teaching approaches that reflect real-life clinical situations.

In this study, logbooks, written exams and clinical assessments were common for assessment, but OSCEs were rarely used. Scenario-based learning, role-playing and simulation-based training have become more popular but are still underused in some regions. As medical education advances, blended learning, combining digital and hands-on training, may become essential for improving ID training worldwide [14].

In many European countries, a PhD is required for academic advancement for those who wish to pursue a scientific and academic career as scientists and/or professors at colleges and universities. In our study, only 34.5% of the countries reported a PhD requirement for academic career. These findings support the previous research showing that PhD requirements in medical specialties vary globally and may affect career mobility [8].

This study revealed significant differences in the number of ID specialists and training centers across countries. Specialist numbers ranged from 10 to over 7200, training centers from none to over 80 in Türkiye and Russia. Limited ID training capacity reflects the growing need for qualified specialists. Low ID training capacity signals an increasing need for specialists. In some countries, current resources are insufficient. Investment in training and retention is essential, especially in low-capacity settings. Addressing global gaps needs a collaborative effort for fair ID training and a stronger workforce.

Our study found that, following the pandemic, ID training programs had high occupancy rates, above 85% in many countries and 100% in some. The perceived importance of the ID specialty was rated at an average of 3.5 out of 5 across the representatives of the participating countries; notable variation was observed in Iraq and Malaysia reporting the highest rating (5/5). The COVID-19 pandemic highlighted the importance of ID specialists in managing infections, guiding treatment and developing vaccines. However, the heavy workload and stress may explain lower post-pandemic ratings in some settings [18].

This study has several strengths. Unlike previous surveys (e.g. ESCMID/UEMS), it includes 29 countries across diverse regions as Europe, the Middle East, Central and South Asia, providing a broader and more diverse snapshot of global ID training systems. National-level data were provided by qualified ID educators directly involved in postgraduate training, ensuring contextual accuracy. This study explores comprehensive aspects of ID training, from curriculum and clinical rotations to assessments and subspecialties, and incorporates up-to-date insights on training preferences and challenges in the post-COVID context.

However, this study has a few limitations that should be noted. The study relied on expert responses from a single respondent per country, which may not reflect all national or local variations. Participation was based on availability within a professional network, which may affect generalizability. The data collection tool was designed for exploratory descriptive use formal validation was not considered necessary.

The observed variability in ID training underscores the essential need for harmonized international standards; structural revisions or recruitment strategies may be warranted in countries with low trainee-to-center ratios; adoption of formal curricula and licensing frameworks could enhance training consistency; increased recognition of ID post-COVID presents an opportunity for systemic improvement; cross-border fellowships and collaborative initiatives may support capacity building in resource-limited settings.

## Conclusion

By including a broader range of countries, this study provides an updated and comprehensive overview of ID specialty training, highlighting regional disparities in structure, duration, curriculum, and workforce capacity, often overlooked in Europe-centered analyses. The findings emphasize the need for harmonized training standards to ensure baseline competence and support cross-border professional mobility, while also allowing flexibility to adapt to local epidemiological needs. Given the global nature of infectious diseases, international collaboration and inclusive training policies are essential to building a resilient and responsive ID workforce.

## Supplementary Information

Below is the link to the electronic supplementary material.

Supplementary file1 (PDF 704 KB)

Supplementary file2 (PDF 274 KB)

## Data Availability

An anonymized version of the data that support the findings of this study is available from the corresponding author upon reasonable request.
