# Supplementary material for: Bridging continents: postgraduate infectious diseases training programs from central Europe to Southeast Asia
Source: Infection. 2025 Jul 8;53(6):2565–85. doi: 10.1007/s15010-025-02597-7 (PMC12675560; doi:10.1007/s15010-025-02597-7)
Supplement: Supplementary file 1 — Supplementary file1 (PDF 704 KB) [file 15010_2025_2597_MOESM1_ESM.pdf]

# Data Collection Tool

**Infectious Diseases Specialty Training Questionnaire**

Dear Participant,

Thank you for participating in this international survey on Infectious Disease (ID) specialty training. The purpose of this study is to explore and compare national-level structures, curricula, and policies of specialty training in Infectious Diseases. This form does not request any personally identifiable or sensitive information. All responses are anonymous and will be used in aggregated form for research purposes only. Participation is completely voluntary and consent is implied by continuing. The first section will collect general professional background information to help contextualize the responses. No personal, private or institutional data will be collected or shared. Thank you for your cooperation in completing the questionnaire.

## Part 1 . Personal Background

This section is intended to collect general professional background information to help contextualize the responses.

**Country of Practice:**

**Academic/Professional Title**:

**Primary Specialty:**

## Part 2. General Information About ID Practices and ID Specialty Training

Please provide general information about ''ID Practices'' and ''ID Specialty Training'' in your country. If you are unable to answer or not sure about the answers, please indicate so or seek information from the relevant authorities of your institute/representatives.

1. **What is the authorized name of *Infectious Diseases Specialty* in your country?**

2. **Which expression is suitable for ID Speciality Training in your country?**

a. Can be entered directly after medical school
b. Can be entered after another specialty training

3. **Is ID an independent speciality in your country, or is it pursued after completing another speciality? Please select the option that describes the structure of ID training in your country.**

a. It is an independent specialty
b. ID is a specialty following internal medicine speciality
c. ID is a speciality following medical microbiology speciality
d. Other (Please specify)…………………………………………………………………..

4. **What is the duration of ID Speciality in your country?**

5. **Does ID have any subspecialties in your country? Please select from the list. (Select all that apply)**

   a. Intensive care
   b. Epidemiology
   c. HIV and AIDS
   d. Immunology and Allergy
   e. Microbiology
   f. Virology
   g. Parasitology
   h. Other (Please mention)……………………………
   i. No subspecialities exist

6. **If there is a mandatory written exam to enter speciality training in your country, which one is suitable from the following list, please select.**
   a. Exam by national authorities. Please write the name of the exam made by national authorities of your country……………………………………………………………………………………
   b. Exam by local authorities
   c. Each institution makes its own
   d. No written exam is needed

7. **How is the Infectious Diseases (ID) specialty ranked in terms of preference (on a scale from 1 to 5) compared to other medical specialties in your country?** (Please base your response on official data or general trends, such as application rates or the number of available positions filled) (1= Least preferred, 3= Moderately preffered, 5= Highly Preffered)

8. **Please score the general expression (1-5); "The importance of ID specialty increased after COVID pandemic in my country" .** (Please base your response on official data or general trends, such as application rates or the number of available positions filled. 1 = No perceived increase in importance, 3 = Moderate change, 5 = Clearly perceived increase in importance)

9. **Please indicate the occupancy rate (%) of the available training positions for ID in the most recent speciality exams in your country**. If possible, provide the percentage for the last available year.

10. **What is the total number of ID specialists in your country?**

11. **What is the number of ID training centers in your country?**

12. **Do you have a national ID speciality association/associations? Please name and provide their web sites (Please write only ID and ID &CM associations; exclude hepatitis, etc. associations ).**
   a. Yes we have one……………………….
   b. Yes we have more than one………………..
   c. No, we do not have
   d. Other (Please mention) ……………………………..

13. **Do you have formal assessments** (for a standard grading system to score every student objectively) **during ID specialty training?** (If yes, please specify the types of assessments used (e.g., knowledge-based assessments, workplace-based assessments) and how they contribute to the standard grading system to objectively evaluate students.

14. **Do you use a logbook and/or portfolio for performance evaluation of ID speciality trainees?**

15. **Do you have an annual Professional Development (Progress) Exam for ID speciality trainees?**

16. **Do you have a summative formal exam for completion of ID speciality training? If yes please explain (oral, clinical, laboratory, written, etc.)**

17. **Is it mandatory to submit a specialization thesis for completion of ID speciality training?**

18. **Do postgraduate ID specialists have a chance to continue their academic career after graduation from a speciality or do they have to do a PhD?**

   a. They can continue academic career
   b. They have to do PhD

19. **Is there a mandatory periodic license exam for ID specialists in your country? If you have a periodic licence exam, how often is the exam required for license renewal?**

## Part 3. Information About ID Specialty Training Curriculum

**This part is about ID specialty training curriculum in your country. If you are unable to answer the questions, please indicate so or seek information from the relevant authorities.**

**20. Do you have a formal ID speciality training curriculum?** a. Yes b. No

**21. Which of these specialities are integral parts/rotations of ID training?** (Select all that apply)

a. General Internal Medicine - ………………………months
b. Medical Microbiology - ………………………months
c. Infection Control - ………………………months
d. Sexually transmitted diseases………………………months
e. Travel Medicine - ………………………months
f. Transplantation - ………………………months
g. Immunization - ………………………months
h. Epidemiology………………………months
i. Other. (Please specify the name and duration)…………………………/…………………………months

**22. Please specify the duration of the rotations of ID specialty training.**

**23. Which of these invasive procedural competencies does your ID training include?** (Select all that apply)

a. Needle Aspiration
b. Abscess Drainage
c. Urinary Catheterization
d. Pleural Fluid Drainage
e. Lumbar Puncture
f. Joint Aspiration
g. Endoscopic Interventions
h. None
i. Other. Please mention……………………………………..

**24. What standards or guidelines is the ID speciality training curriculum based on in your country?** (For example, are there national, regional, or international standards that shape the curriculum?)

a. National standards
b. International standards (such as UEMS)
c. Other (Please specify)………………………………

25. **Please select the topics included in your ID specialty training curriculum** (Select allthat apply).

a. Rational antibiotic use
b. Clinical epidemiology
c. Critical care medicine
d. Health economics
e. Healthcare associated infections
f. HIV
g. Immunology
h. Immunization
i. Infection control
j. Laboratory management
k. Palliative care
l. Travel Medicine
m. Wound care
n. Quality improvement
o. Research methodology
p. Team work
q. Communication skills
r. Leadership
s. Other. Please mention…………………………………………..

26. **Please select the training methods used during your ID training** (Select all that apply).

*a. Traditional (Lectures, didactic sessions)*
*b. Practical exercises*
*c. Field training (observation, community based)*
*d. Hands on workshops (simulation practices, skills stations)*
*e. Online and e-learning (web-based courses, interactive modules)*
*f. Mobile apps*
*g. Webinars and virtual conferences*
*h. Printed materials and manuals*
*i. Role playing, scenario based training (standardized and simulated patients, etc.)*
j. Journal clubs
k. Sudy groups
l. Other. Please specify…………………………………………..

27. **Is there a compulsory period of training conducted in other institutions or abroad included in your ID Speciality Training curriculum? * If your answer is "yes" please explain it.**

28. **If there is a national quality assurance/accreditation for your ID Speciality training, provide the name please.**

29. **Is there anything else you would like to add regarding ID speciality training or any related topics?**

***Thank you for your valuable time and contributions to this questionnaire…***
