# Supplementary material for: Bridging continents: postgraduate infectious diseases training programs from central Europe to Southeast Asia
Source: Infection. 2025 Jul 8;53(6):2565–85. doi: 10.1007/s15010-025-02597-7 (PMC12675560; doi:10.1007/s15010-025-02597-7)
Supplement: Supplementary file 2 — Supplementary file2 (PDF 274 KB) [file 15010_2025_2597_MOESM2_ESM.pdf]

Supplement 1. National Infectious Diseases Associations (Verified Name & Website)

Country	National Infectious Disease Association (Verified Name & Website)
**Albania**	Albanian Society of Infectious Diseases
**Azerbaijan**	Azerbaijan Society of Infectious Diseases and Clinical Microbiology - https://www.instagram.com/amic.2022/
**Bosnia and Herzegovina**	Association of Infectiologists in B&H - https://aiubih.org/
**Croatia**	Croatian Society for Infectious Diseases - https://hdib.hr/
**Greece**	Hellenic Society for Infectious Diseases
**India**	Clinical Infectious Diseases Society
**Iran**	Iranian Infectious Diseases Society, Iranian Society of Clinical Microbiology and Infectious Diseases
**Iraq**	Iraqi Board for Medical Specializations - https://www.iraqiboard.edu.iq/ar<br><br>Arabian Board for Health Specializations - https://www.abhs.edu.iq/ar
**Kazakhstan**	Association of Infectious Diseases Doctors
**Kosovo**	Infectious Diseases Association of Kosova
**Kyrgyz**	Republic Association of Infectious Diseases and Hepatologists of Kyrgyzstan
**Lebanon**	Lebanese Society of Infectious Diseases and Clinical Microbiology (Website under construction)
**Malaysia**	Malaysian Society of Infectious Diseases and Chemotherapy - https://www.myicid.com/
**Moldova**	Association of Infectious Diseases Specialists from Republic of Moldova
**North Macedonia**	The North Macedonian Society for Infectious Diseases - https://www.zim.org.mk/
**Pakistan**	MMIDSP (Medical Microbiology and Infectious Disease Society of Pakistan)
**Qatar**	Qatar Infectious Disease Society under Qatar Medical Association
**Romania**	National Society of Infectious Diseases - https://snrbi.org/
**Russia**	National Association of Specialists for Infectious Diseases named after V.I. Pokrovsky
**Saudi Arabia**	Saudi Society of Clinical Microbiology and Infectious Diseases
**Serbia**	Association of Infectious Diseases of Serbia
**Slovenia**	Infectious Diseases Society of Slovenia, Slovenian Society for Antimicrobial Chemotherapy
**Türkiye**	Turkish Infectious Diseases and Clinical Microbiology Specialist Association - https://www.ekmud.org.tr/<br><br>Turkish Society of Clinical Microbiology and Infectious Diseases - https://www.klimik.org.tr/
**United Arab Emirates**	The Emirates Infectious Disease Society -https://www.ema.ae/
